# Quantification of transmural perfusion gradients by high-resolution MR versus fractional flow reserve for the assessment of coronary artery stenosis

**DOI:** 10.1186/1532-429X-14-S1-P5

**Published:** 2012-02-01

**Authors:** Amedeo Chiribiri, Gilion Hautvast, Tim Lockie, Andreas Schuster, Boris Bigalke, Luca Olivotti, Simon Redwood, Marcel Breeuwer, Eike Nagel, Sven Plein

**Affiliations:** 1 Imaging Sciences and Biomedical Engineering, King's College London, London, UK; 2 Imaging Systems - MR, Philips Healthcare, Best, Netherlands; 3 Cardiovascular Division, King's College London, London, UK; 4 Biomedical Image Analysis, Eindhoven University of Technology, Einhoven, Netherlands; 5 Division of Cardiovascular and Neuronal Remodeling, University of Leeds, Leeds, UK

## Background

The subendocardial layer of the myocardium is more sensitive to ischaemia than the subepicaridal layer due to interactions between myocardial contraction and blood supply. The transmural perfusion gradients observed in coronary heart disease (CAD) can be visualised with high spatial resolution myocardial perfusion CMR. These gradients are characterised by the extent and intensity of the endo- to epicardial redistribution and by its temporal persistence. The gradientogram method (Hautvast et al. MRM 2011) has been developed to assess and quantify these characteristics of transmural perfusion gradients. The aim of this study was to assess the diagnostic accuracy of the method versus fractional flow reserve (FFR) in patients with suspected CAD.

## Methods

28 patients (20 male, 58±10 years) with known or suspected CAD underwent high-resolution (1.2 x 1.2 mm in plane) adenosine stress perfusion CMR at 3.0T. FFR was measured in all vessels with >50% severity stenosis. FFR<0.80 was considered hemodynamically significant. Transmural perfusion gradients were measured by the gradientogram plot and initially analysed based on different thresholds of transmural perfusion redistribution. In addition, the following parameters were assessed (units of measurement): radial extent (degrees), peak value (% of maximum transmural gradient), area (degrees*seconds), temporal persistence (seconds) and strength of the gradient (% of transmural gradient * seconds-1).

## Results

Transmural perfusion gradient analysis alone at thresholds of 5%, 10%, 15% and 20% yielded a diagnostic accuracy for CAD of 0.72, 0.78, 0.83 and 0.78, respectively (Table 1). Adding the quantitative gradient parameters to the analysis resulted in an improved maximum diagnostic yield. The combination of the temporal persistence of subendocardial perfusion defects with a 20% threshold of gradient amplitude showed the highest diagnostic yield with 0.91 AUC, sensitivity 0.8, specificity 1. The second best combination of parameters was the area of the gradient (defined as radial extent multiplied by temporal persistence) at a threshold of 20% that also yielded a sensitivity of 1 and a specificity of 0.8 with an AUC of 0.88.

**Table 1:** Diagnostic accuracy of gradientogram analysis (AUC on ROC analysis) by simple thresholding and by adding to the analysis several quantitative parameters related to the transmural perfusion gradients

	Gradient threshold 5%	Gradient threshold 10%	Gradient threshold 15%	Gradient threshold 20%
Gradient yes/no (without considering any quantitative measurements)	0.72	0.78	0.83	0.78

				

Average gradient extent	0.66	0.84	0.78	0.71
Average gradient peak	0.74	0.83	0.71	0.74
Average gradient area	0.68	0.85	0.79	0.88
Average gradient amplitude	0.65	0.84	0.85	0.76
Average gradient persistence	0.76	0.72	0.72	0.91
Average gradient strength	0.73	0.84	0.83	0.85

## Conclusions

Quantification of transmural perfusion gradients based on derived measurements from the gradientogram plot allows an accurate diagnosis of hemodynamically significant CAD as compared to FFR. Additional derived measures increase the diagnostic yield over simple thresholding, in particular the temporal persistence of a perfusion gradient or a combination of the radial extent and temporal persistence (area of the gradient).

## Funding

The Centre of Excellence in Medical Engineering funded by the Wellcome Trust and the Engineering and Physical Sciences Research Council (EPSRC) under grant number WT 088641/Z/09/Z. Andreas Schuster received grant support from the British Heart Foundation (BHF) (RE/08/003 and FS/10/029/28253) and the Biomedical Research Centre (BRC-CTF 196). Sven Plein was funded by a Wellcome Trust Research Fellowship during the early phase of this study (WT078288).and is currently funded by a British Heart Foundation Senior Research fellowship (FS/10/62/28409). Timothy Lockie was funded by a British Heart Foundation Clinical Research Training Fellowship (FS/08/058/25305).

**Figure 1:**
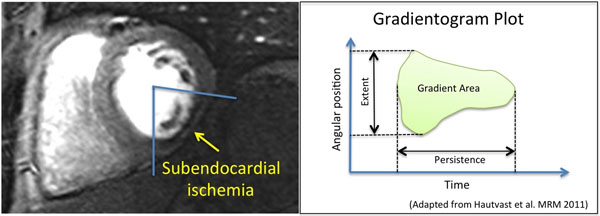
Left: example of a patient showing inferior subendocardial ischemia. Right: the gradientogram plot allows measurements of radial extent, temporal persistence, area, peak and average intensity as well as strength of the transmural perfusion gradient (see the text for details on the units of measurements).

